# Treatment-resistant depressed patients show a good response to Mindfulness-based Cognitive Therapy

**DOI:** 10.1016/j.brat.2006.04.008

**Published:** 2007-03

**Authors:** M.A. Kenny, J.M.G. Williams

**Affiliations:** a The Adelaide Clinic Consuting Suites, 33 Park Terrace, Gilberton, South Australia 5081, Australia; b University Department of Psychiatry, University of Oxford, UK

**Keywords:** Mindfulness, MBCT, Depression, Treatment resistance

## Abstract

Mindfulness-based Cognitive Therapy (MBCT) is a class-based programme designed for use in the prevention of relapse of major depression. Its aim is to teach participants to disengage from those cognitive processes that may render them vulnerable to future episodes. These same cognitive processes are also known to maintain depression once established, hence a clinical audit was conducted to explore the use of MBCT in patients who were currently actively depressed, and who had not responded fully to standard treatments. The study showed that it was acceptable to these patients and resulted in an improvement in depression scores (pre-post Effect Size=1.04), with a significant proportion of patients returning to normal or near-normal levels of mood.

## Introduction

The emotional, social and economic burden of depression for sufferers, their families and society is significant, with 12 month prevalence rates estimated at 2.9–12.6% and lifetime risk estimated at 17–19% (Kessler et al., 1994). The fact that depression is often a chronic relapsing condition, with relapse rates of 50–80% in those who have been depressed before (Judd, 1997) has contributed to the WHO prediction that, by 2020, depression will be the second biggest contributor to ill-health burden world-wide (Murray & Lopez, 1998). Individual CBT has been shown to be effective at treating acute depression and reducing relapse (DeRubeis et al., 2005; Hollon et al., 2005) but waiting lists for individual therapy are lengthy in most healthcare settings.

Even treatment by normally ‘effective’ means leaves a substantial minority failing to meet criteria for remission. In a trial to examine relapse risk in the 12 months following antidepressant versus cognitive treatment, Hollon et al. (2005) found that, of the patients recruited for the first (acute treatment) phase, only 58% of the ADM group and the same proportion of the CT group met predefined criteria for recovery and were able to proceed to the relapse phase of the study. The fact that around 40% did not meet such criteria following an adequate ‘dose’ of treatment show that there is still a great deal to do to help those patients who are often called ‘treatment resistant’.

Moreover, depression has another feature that is a considerable cause for concern: the development of a chronic clinical course, which also resists treatment. In these cases, patients report continuing symptoms of depression and accompanying distress about these symptoms. Some 15–39% of cases still meet criteria for Major Depressive Disorder (MDD) 1 year after symptom onset (Berti Ceroni, Neri, & Pezzoli, 1984; Van Valkenburg, Akiskal, Puzantian, & Rosenthal, 1984), and 22% of cases may continue to do so up to 2 years later (Keller, Lavori, Lewis, & Klerman, 1983). Of particular concern is the risk of suicidal behaviour in such patients. One in seven patients hospitalised for major depression die by suicide (Powell, Geddes, Deeks, Goldacre, & Hawton, 2000) and the Population Attributable Ratio (PAR) for depression in serious but non-fatal suicidal behaviour (that proportion of suicidal behaviour that would be removed if depression were taken out of the picture) is 80 per cent (Beautrais et al., 1996). If suicidal ideation occurs during one episode of depression, it tends to recur in later episodes (Williams, Crane, Barnhofer, van der Does & Segal, 2006), making the question of how best to treat recurrent and persistent depression particularly urgent for such at-risk patients.

What is it that keeps people depressed? Nolen-Hoeksema's Responses Styles Theory (1991) suggests that people who engage in ‘repetitive and passive thinking about one's symptoms of depression’ tend to prolong the very symptoms they are trying to reduce. Ruminators often hold positive (but erroneous) beliefs that it will help, not realising that they are reducing their capacity to effectively problem-solve (Watkins & Moulds, 2005). Evidence suggests that the cognitive processes that increase vulnerability to future episodes are the same as those that maintain depression. These processes are depressive rumination (Jacobson, Martell & Dimidjian, 2001; Nolen-Hoeksema, 1991, 2000; Watkins & Teasdale, 2004) and high cognitive reactivity to mood shifts, where the experience of low mood more easily triggers negative thinking in previously depressed patients (Segal, Gemar, & Williams, 1999; Teasdale, 1999).

Mindfulness-based Cognitive Therapy (MBCT) arose out of an investigation into the cognitive processes that render depressed individuals vulnerable to repeated relapse and recurrence (Teasdale, Segal, & Williams, 1995) with a view to offering a programme that would target those cognitive vulnerabilities (Segal, Williams, & Teasdale, 2002). The intervention was designed as a class-based intervention to increase accessibility to effective relapse prevention. It incorporates, as a central component, mindfulness training as developed by Kabat-Zinn and his colleagues at the University of Massachusetts Medical Center (Kabat-Zinn, 1990), and adds components of cognitive behaviour therapy for depression (Beck, Rush, Shaw, & Emery, 1979). Two studies have demonstrated this group programme's efficacy in reducing relapse rates of depression at 12 month follow-up compared to treatment as usual (Ma & Teasdale, 2004; Teasdale et al., 2000).

MBCT teaches participants to observe their thoughts and feelings through the repeated practise of intentionally returning attention to a neutral object (e.g. the breath or body sensations) in the present moment. Participants are taught how to cultivate direct experiential awareness, together with an attitude of non-judgmental acceptance, towards whatever is present (including sad mood, which in previously depressed patients is likely to trigger patterns of global negative self-referent thinking). The cultivation of awareness during mindfulness practise enables patients to see more clearly when negative and ruminative responses are being triggered, and allows them to decentre from such patterns of thought, seeing them as mental events, rather than necessarily valid reflections of reality. Unlike standard CBT, where the focus is on changing the content of thoughts, MBCT's focus is on fostering meta-cognitive awareness and the modification of meta-cognitive processes that maintain unhelpful reactive or ruminative mind states. So although MBCT was designed for patients in remission, the above analysis suggests that it might be helpful for symptomatic patients who remain caught in such unhelpful thought processes.

The present article describes a clinical audit of a consecutive series of patients referred for MBCT, focussing on those who had continuing symptoms of depression despite treatment with antidepressant medication (ADM) or CBT or both. The two randomised controlled studies conducted on MBCT had shown that those with three or more episodes had a significant reduction in relapse rates but that those with two or less had a non-significant trend towards an increased risk of relapse in the year of follow-up (Ma & Teasdale, 2004; Teasdale et al., 2000). The inclusion criteria for the MBCT programme therefore retained the three-or-more-episodes feature for those who had been recurrently depressed, but was expanded to include patients whose mood disorder was running a chronic course, where it was hypothesised the depression was being maintained by a similar ruminative cognitive style.

The audit was conducted to examine the effects of this approach with this group, the acceptability of the class format, and the feasibility of including both recovered and symptomatic patients in the same classes. It was hypothesised that the class format, with a mix of recovered and symptomatic patients, would be acceptable as the meta-cognitive approach taken means there is no need to work with the unique content of each individual's automatic thoughts, whether currently negative in tone or not.

There are compelling reasons to wish to check out the possible relevance of MBCT for patients who remain in episode. The developers of MBCT have been very cautious about using MBCT for such patients. ‘It is important to note that MBCT was specifically developed for remitted patients and is unlikely to be effective in the treatment of acute depression, where factors such as difficulty in concentrating and the intensity of negative thinking may preclude acquisition of the attentional control skills central to the programme’ (Teasdale et al., 2000, p. 622). However, since a considerable proportion of the patients in this study had already received CBT, we wished to examine patients’ responses to an approach that might represent a back-up procedure if the currently available empirically supported treatments (ESTs) such as CBT and anti-depressant medication fail.

Finally, we were particularly interested in those patients who had suicidal ideation as part of their symptoms of depression. We wished to examine the hypothesis that such patients might respond significantly *less* well to MBCT. Such patients are known to be highly reactive to depressed mood and to react to small mood shifts with impaired problem solving (Williams, Barnhofer, Crane, & Beck, 2005), and we were concerned that this pattern might undermine the ability or motivation to practise mindfulness.

## Method

### Participants

Seventy-nine patients participated in eight consecutive MBCT programs, which ran consecutively over a two and a half year period through a CBT clinic. The first author (MK), a psychiatrist and cognitive therapist, led the classes. They were aged between 17 and 61 years, 20 were male and 59 were female, and all but one were tertiary referrals, having been treated in general practise and by a psychiatrist before referral for MBCT. The exception was a male patient who had only ever seen a GP with a special interest in mental health for his management, and who was recommended for the course by his wife who had previously completed it.

The inclusion criteria for entry into the MBCT programme were that participants had to meet DSM IV criteria for MDD, Bipolar Affective Disorder (BPAD), Depressed phase, or Dysthymia. Where participants had major depression, they also had to have had either three or more episodes of depression or have had a chronic course of greater than 1 year following a major depressive episode that appeared, at clinical interview, to be related to the presence of ruminative thought patterns. Participants had to be prepared to practise between classes and to attend all classes, and therefore be open to using meditation as a way of managing their condition once the rationale was explained at the pre-course interview. The exclusion criteria were that they could not be abusing substances in a way that would interfere with being able to meditate in clear consciousness. Suicidal patients were not excluded unless they were at immediate risk and were without a monitoring therapist. If their external therapist was prepared to monitor this and liase as necessary with the MBCT instructor, the participant could proceed.

### Demographic and diagnostic information for the audited participants

The clinical audit was conducted only on those patients (*N*=50 out of 79) who were (a) still symptomatic at the start of the MBCT course, symptomatic being defined as the presence of continuing symptoms assessed by clinical interview and a Beck Depression Inventory (BDI)>10, and (b) met DSM IV criteria for either MDD or BPAD, depressed phase.

All the diagnoses had been confirmed by at least one psychiatrist during the referral and selection period. One participant became an in-patient during the course. All others were outpatients. Two other patients were symptomatic at the start of the course but as they only met criteria for Dysthymia, they were not included in this audit.

The mean age was 43.3 (s.d.=9.7, range 17–61), with 37 women (74%). The majority of the sample (90%) were Australian born, and 31 (62%) were married/in defacto relationships, 11 (22%) were single and eight (16%) divorced. In terms of employment and educational status, 30 (60%) were tertiary educated, 36 (72%) were employed, but of the unemployed group (14), 10 (71%) were on disability pensions due to their psychiatric conditions. Diagnostically, 45 had MDD, three had BPAD I, and two had BPAD II, and were in the depressed phase. Thirty-nine of the MDD patients had experienced three or more episodes, and six were experiencing a current episode lasting more than 1 year. Four of the BPAD depressed patients had three or more episodes, and one had a chronic episode lasting 6 years. The mean duration of chronic depression in the whole group was 10.11 years (s.d.=8.01, range 1.25–20 years). Table 1 shows the percentage of patients reporting each symptom of depression.

### Other treatments

Since the MBCT was done as part of routine clinical practise, no attempt was made to prevent referring clinicians from changing ADMs or changing the dose of existing ADMs. Thirty-seven of the 50 (74%) were on antidepressant medication at the start of the course, one patient had her dose reduced during the course, and six had their medication changed or increased. Thirty-four (68%) had received a complete course of CBT from an experienced cognitive therapist (30 within the last 5 years, four in the past 10 years) prior to attendance for MBCT. None were receiving CBT during the MBCT programme.

### MBCT treatment

All participants had an individual interview before the classes started to assess suitability and prepare them for the course. The course followed the MBCT programme as described by Segal, Williams, and Teasdale (2002). Eight 2-h classes were held, up to an hour of which is spent in meditation practices. Each class had up to 14 participants. Participation in class discussion was voluntary. Homework involved around 1 h per day of meditation or yoga, and other related formal and informal practices for the 8 weeks. An individual session was offered to all participants after the course ended to discuss progress, plan ways to act on the relapse prevention plan, and continue the meditation practise if the latter was found helpful. Ongoing follow-up was also arranged if found to be required.

### Measures

The audit used pre and post BDIs as its measure of outcome. In addition, all participants were asked to give a rating of how important the MBCT group had been to them using a Likert scale from 0 (not at all important) —10 (extremely important).

## Results

Of the 50 patients audited, 49 completed the programme (99%), and pre and post questionnaires were completed by 46 of these completers (94%).

### Acceptability of MBCT

At the end of the MBCT programme, patients were asked to rate how important the programme had been to them. The mean rating (max=10) was 8.5 (1.5). 83% rated it 7 or over. Only two patients out of the 79 dropped out of MBCT, and only one out of the 50 depressed patients.

### Effect of MBCT on Depression (BDI) scores

The mean BDI score prior to MBCT was 24.3 (s.d=9.8, range 11–46). The mean BDI score following MBCT was 13.9 (s.d=9.7, range 1–41). This represents a highly significant change (*t* (47)=6.01; *p*<0.0001). We also calculated the intraclass correlation (ICC) to take account of the non-independence of data from members of the same group (Baldwin, Murray, & Shadish, 2005), and found it to be 0.001. The adjusted *t* scores taking account of the ICC remained significant (*t* (47)=5.98) and the *t* score remains significant if the degrees of freedom are based (conservatively) on the number of groups rather than the number of participants (*p*<0.001).

Before treatment, 14 patients had scores of 30 or above. After treatment, only four patients had a score over 30. Whereas before treatment, no patient had a score below 11, now 20 of the 46 patients (43%) scored below 10 and a further eight scored below 14 (that is, 61% of the sample had a post-treatment score below 14). Cohen's ‘*d*’ statistic was computed using pooled standard deviation, and yielded an Effect Size of 1.04 for pre minus post-test change.

**Fig. 1:**
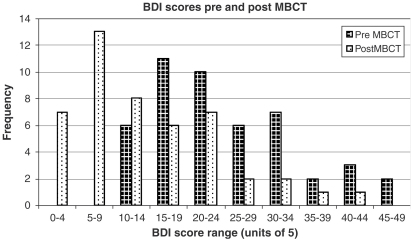
BDI scores pre and post MBCT.

Of the 47 patients with complete data, four became worse (BDI scores rising from 25–29, 14–18, 15–21, and 11–32). Using the Jacobson and Truax (1991) formula for computing meaningful change, two of these patients crossed the cut-off score of 19.1. Post-treatment interviews were conducted with each patient to establish the reason for this increase, and in each case the patient claimed it was part of the familiar mood fluctuations they experience in response to adverse life events (e.g. job pressures, relationship conflict, etc.). These reports must be treated cautiously, however, since the instructor of the classes conducted the interviews. Consistent with these reports, however, all four patients rated the MBCT programme as highly important to them, with scores (max=10) 9, 8, 7 and 9, respectively (Fig. 1).

### Effect of severity of pre-treatment depression

To examine whether response to MBCT depended on initial level of severity of depression, we divided the group by the pre-test median (24) to form a ‘moderate’ (BDI 24 or below) and a severe (BDI 25 or above) group. A Severity×Time ANOVAR yielded a significant Main Effect for Time, as expected, and Mean Effect of Severity (*F* (1, 44) 39.1, *p*=0.0001) and a significant interaction of Time and Severity (*F* (1, 44)=13.8, *p*=0.001). The ‘moderate’ group (*N*=26) fell from a pre-test BDI of 17.2 (s.d.=4.3) to a post-test of 11.5 (s.d.=7.8), a pre-post Effect Size of 0.9); while the ‘severe’ group (*N*=24) reduced their BDI scores from 33.2 (s.d.=6.4) to a post-test of 17.1 (s.d.=11.2), a pre-post Effect Size of 1.8.

### Effect of suicidal symptoms on response to MBCT

At pre-test, diagnostic information (Table 1) showed that 32 reported thoughts of death and suicide. We compared the pre-treatment BDI scores for these patients with those who did not report such thoughts. The mean BDI for suicidal patients was 27.2 (10.6) before treatment, which was significantly higher than the scores for patients without suicidal thoughts (*M*=20.2; s.d.=5.7; *t*(49)=3.16, *p*=0.003).

We examined which BDI items significantly differed at pre-treatment for those who did and those who did not report suicidality. As expected, Item 9 (*suicidality*) differed most significantly (a validation of the clinical diagnostic information). However, four other items also differed significantly (Item 1, *sadness*; item 4, *lack of pleasure*; item 5, *guilt*, and item 12, *loss of interest*).

Following treatment, both groups declined in their scores (post-treatment BDI: suicidal *M*=14.8, s.d.=10.6; non-suicidal *M*=12.8, s.d.=7.8, *t*=0.72, ns). A Group (suicidal vs. non-suicidal)×Time (pre vs. post treatment) analysis of variance yielded a main effect for Time (*F* (1,46)=37.96; *p*<0.0001), a near significant effect for Group (suicidal vs. non-suicidal) (*F* (1,45)=3.7; *p*=0.06). The Group×Time interaction was not significant (*F*=2.24, *p*=0.141). This pattern of data indicates that whereas suicidal individuals began MBCT with a greater severity of depression, there were no differences in their pattern of response to MBCT.

### Bipolar Affective Disorder (BPAD)

Five patients entered MBCT in the unremitted depressive phase of bipolar disorder. All their BDI scores reduced, falling from 32 to 22, 45 to 6, 15 to 9, 12 to 3, and 17 to 5 at pre-treatment and post-treatment, respectively.

### Effects of other treatments

We examined whether having had prior CBT made a difference to these outcomes, which it did not. The Main Effect for CBT vs. no CBT (*F*(1,44) <1) was non-significant, and the interaction between CBT and Time was also non-significant (*F*<1).

Six patients had medication increased or changed during the 8 weeks of the programme. Reanalysis of the data excluding these patients made no difference to the results (pre-treatment BDI, *M*=24.2, s.d.=9.9; post-treatment BDI, *M*=13.0, s.d.=9.7; *t* (39)=5.6, *p*<0.0001). An ANOVAR taking account of those who remained stable compared to those who changed type or dose of medication, yielded only a significant Main Effect for use of antidepressants (*F* 1, 44)=4.5, *p*=0.04) but no significant ADM×Time interaction. The Main Effect was due to the generally higher scores at both pre and post test for those on medication (pre: *M*=26.5, s.d.=10.0; post: *M*=15.3, s.d.=10.3 for patients on medication vs. pre *M*=20.2, s.d.=7.3; post, *M*=10.4, s.d.=6.9 for those not taking medication).

## Discussion

The results of this preliminary audit suggest that MBCT is an acceptable treatment for patients who have only had a partial response to antidepressant medication and/or standard individual CBT. Further, for many patients it appears to be effective in significantly reducing levels of depression, even in those who start with a more severe pattern including suicidal depression. Although the numbers with bipolar depression are small (*n*=5), it appeared to have an effect in these patients also. Finally, MBCT proved to be an acceptable and useful treatment for one in-patient who was hospitalised due to a worsening of agitated depression just after the course began (BDI falling from 40 to 19).

The overall pre-post Effect Size was large, though smaller than that which would have been expected for pre-post CBT for naïve patients (those who have never had CBT before). In this case, we might expect a pre-post Effect Size of 2.2 (Ost, 2006). It is of note, however, that the proportion achieving remission was equivalent to a recent trial of 16 weeks of CBT (58%; DeRubeis et al., 2005), and that, for the more severe patients, the pre-post Effect Size (1.8) was of the same order of magnitude for individual CBT for naïve patients. Furthermore, in Baer's review of Mindfulness-based Interventions (Baer, 2003), the average Effect Size was 0.71 for pre-post change across a range of physical and psychiatric disorders, and 0.90 where depression was included as a dependant measure. The outcomes in the present study were comparable to these previous reports.

A number of methodological limitations need to be considered here. Firstly, the fact that there is no control group means that that the benefits (and setbacks) seen in the participants cannot be conclusively assigned to the treatment. Secondly, these were patients whose treatment had not proved as effective as either they or their therapists had wished; it is therefore possible they were a highly motivated group. This means that MBCT may still not be effective as a front-line treatment for depression. Thirdly, there was no systematic data collected for these patients on other Axis I and Axis II disorders, their response to past treatments or their openness to non-traditional treatments that, in other settings, may be associated with effectiveness of pharmacological and psychological treatment.

Finally, four patients became worse in the BDI scores, and although they claimed that the programme was important to them, and that the rise in BDI scores was due to their normal mood fluctuations, further research needs to take account of the possibility of adverse effects. Overall though, the concern of Teasdale et al. (2000) that MBCT was unlikely to be effective in the treatment of acute depression was not borne out, even though 98% of patients identified problems with concentration and energy at the outset of the study. In addition, the argument that the sedentary focus of many of the practices (i.e. sitting meditation) may be de-activating and thus counter-productive for such patients again was not borne out. While, at times, some flexibility about the length of the meditation practices was required, participants were expected to and were mostly able to manage the 45 min as long as they learned to bring a kindly and non-judgemental attitude to this, which was repeatedly emphasised by the instructor. The yoga component of the course may also have acted to counteract the cycle of inactivity or agitation frequently encountered in depression.

If these results can be replicated, they may be seen to be consistent with the theoretical changes taking place within and outside cognitive theory and therapy that lay greater emphasis on the processes underlying cognition rather than its content (Hayes, Follette, & Linehan, 2004). The findings are similar to those in another study which examined the effects of mindfulness meditation on depression (Ramel, Goldin, Carmona, & McQuaid, 2004), and fit with the hypothesis that the processes (increased cognitive reactivity and ruminative responses), which play a role in causing relapse of depression, also play a role in the maintenance of depression once it has been established. Targeting these processes with an intervention like MBCT may then have an adjunctive role where standard treatments have failed to bring about a full therapeutic response.

In summary, we found that MBCT was associated with reductions in depression symptoms, with a large pre-post Effect Size (*d*=1.04). Even given the amount of time and commitment involved, the intervention had a high degree of acceptability and low dropout rate, despite the levels of severity as well as the disabling effects of numerous prior episodes of depression. Further research into the use of MBCT in the active phase of treatment of recurrent or chronic depression is indicated, given the promising results described, and the potential economic advantages of being able to offer this in a group format to a mix of both symptomatic and recovered patients.

## Figures and Tables

**Table 1:** Symptoms of depression reported by 50 treatment-resistant patients

Symptom	Number	Percentage
Depressed mood	44	86
Loss of interest	49	96
Appetite/weight change	35	70
Sleep disturbance	45	90
Psychomotor changes	45	88
Loss of energy	50	98
Worthlessness/guilt	46	92
Diminished concentration	49	98
Thoughts of death/suicide	32	63
